# FabR regulates *Salmonella* biofilm formation via its direct target FabB

**DOI:** 10.1186/s12864-016-2387-x

**Published:** 2016-03-22

**Authors:** Kim Hermans, Stefanie Roberfroid, Inge M. Thijs, Gwendoline Kint, David De Coster, Kathleen Marchal, Jos Vanderleyden, Sigrid C. J. De Keersmaecker, Hans P. Steenackers

**Affiliations:** Department of Microbial and Molecular Systems, Centre of Microbial and Plant Genetics, Katholieke Universiteit Leuven, Kasteelpark Arenberg 20, 3001 Leuven, Belgium; Platform Biotechnology and Molecular Biology, Scientific Institute of Public Health (WIV-ISP), Brussels, Belgium

**Keywords:** *Salmonella*, Biofilm, FabR, FabB, Unsaturated fatty acids, ChIP-chip

## Abstract

**Background:**

Biofilm formation is an important survival strategy of *Salmonella* in all environments. By mutant screening, we showed a knock-out mutant of *fabR*, encoding a repressor of unsaturated fatty acid biosynthesis (UFA), to have impaired biofilm formation. In order to unravel how this regulator impinges on *Salmonella* biofilm formation, we aimed at elucidating the *S*. Typhimurium FabR regulon. Hereto, we applied a combinatorial high-throughput approach, combining ChIP-chip with transcriptomics.

**Results:**

All the previously identified *E. coli* FabR transcriptional target genes (*fabA*, *fabB* and *yqfA*) were shown to be direct *S.* Typhimurium FabR targets as well. As we found a *fabB* overexpressing strain to partly mimic the biofilm defect of the *fabR* mutant, the effect of FabR on biofilms can be attributed at least partly to FabB, which plays a key role in UFA biosynthesis. Additionally, ChIP-chip identified a number of novel direct FabR targets (the intergenic regions between *hpaR*/*hpaG* and *ddg*/*ydfZ*) and yet putative direct targets (*i.a.* genes involved in tRNA metabolism, ribosome synthesis and translation). Next to UFA biosynthesis, a number of these direct targets and other indirect targets identified by transcriptomics (e.g. ribosomal genes, *ompA*, *ompC*, *ompX*, *osmB*, *osmC, sseI*), could possibly contribute to the effect of FabR on biofilm formation.

**Conclusion:**

Overall, our results point at the importance of FabR and UFA biosynthesis in *Salmonella* biofilm formation and their role as potential targets for biofilm inhibitory strategies.

**Electronic supplementary material:**

The online version of this article (doi:10.1186/s12864-016-2387-x) contains supplementary material, which is available to authorized users.

## Background

Bacteria predominantly grow inside multicellular communities attached to solid surfaces and enclosed in a self-produced polymeric matrix, called biofilms [1]. In fact, it was shown that the majority of all bacterial infections are related to biofilm growth, stressing the importance of this life style [2]. Because of the protected environment, bacteria within biofilms are less sensitive to environmental stresses, including disinfectants and antibiotics, and are as a consequence very difficult to eradicate [3]. Pathogens like *Salmonella* are able to survive in biofilms on biotic as well as abiotic surfaces [4] as was shown for *i.a.* plastic, stainless steel, plant surfaces and gallstones [3, 5–7].

The extracellular matrix of *Salmonella* biofilms contains a variety of proteinaceous compounds and exopolysaccharides, including curli fimbriae and cellulose [8]. Furthermore, the presence of flagella and fatty acid containing structures such as lipopolysaccharides was shown to be important in *Salmonella* biofilms [7]. However, the exact composition of the matrix and the appearing ratios of the different structures are highly dependent on the environmental conditions in the used biofilm set-up [9]. It was shown for example that an incomplete LPS fraction does not affect biofilm formation capacity of *Salmonella* on hydrophobic gallstone surfaces, but highly reduces its biofilm capacity on hydrophilic glass surfaces [7].

Synthesis of all these structures is strongly regulated as the regulatory networks inside biofilms as well as metabolism are highly complex [4]. The central transcription regulator in *Salmonella* biofilm formation metabolism is CsgD, which positively regulates the production of curli and cellulose in the extracellular biofilm matrix [10, 11]. This regulator itself shows an enhanced expression in the presence of high c-di-GMP concentrations [12], a secondary messenger molecule which has been studied extensively in regulation of bacterial multicellular behavior, motility and virulence [13]. Knowledge about the complex regulatory processes in biofilm formation can provide more insight into survival strategies of *S.* Typhimurium in non-host environments and can be the fundament of new eradication methods.

Bacteria strictly regulate their cellular membrane composition in response to changes of environmental conditions, in order to adjust membrane fluidity and optimize associated membrane functions [14]. Variations in growth temperature [15–18], pH [16, 19, 20], ethanol concentration [21] and external osmolality [22], as well as transition to the stationary phase [23] have been shown to lead to changes in membrane fatty acid composition. These changes in membrane fatty acid composition and membrane fluidity have been shown to affect bacterial thermotolerance [24, 25], acid resistance [26] and pressure resistance [27] .

In *Escherichia coli*, a mechanism was discovered that controls the ratio of mono-unsaturated fatty acids (UFA) compared to saturated fatty acids (SFA) present in the membrane in response to the composition of the cellular pool of long chain acyl-thioesters. [28]. A central transcription regulator in this process is FabR (also called YijC) which, in response to mono-unsaturated fatty acid thioesters, possesses an enhanced affinity for binding to the promoter sequence of *fabB* and in lesser extent to the *fabA* promoter. This promoter binding represses transcription of these *fab* genes which are involved in UFA biosynthesis [29]. FabA introduces the double bond into the growing acyl chain by catalyzing dehydration of β-hydroxydecanoyl-ACP and the isomerization of the resulting product to cis-3-decenoyl-ACP [29]. FabB elongates cis-3-decenoyl-ACP to cis-5-dodecenoyl- ACP, which enters the standard fatty acid synthesis cycle and becomes elongated to the 16- and 18-carbon UFAs [30]. Conversely, another transcription regulator, FadR, stimulates UFA biosynthesis by binding to the *fabA* end *fabB* promoter. This activation is relieved by dissociation of FadR from the *fabA* and *fabB* promoter regions in response to long-chain fatty acids [31]. Together these transcription regulators ensure a well-balanced ratio of SFA compared to UFA and as such sustaining the biophysical properties of the cell membrane phospholipids that are of great importance for bacterial growth and survival.

By mutant screening, we found a *fabR* knock-out mutant of *Salmonella enterica* serovar Typhimurium to have an impaired biofilm formation. To get more insight into the way FabR regulates *Salmonella* biofilm formation, we mapped the full *S.* Typhimurium SL1344 FabR regulon and identified its direct and indirect target genes. Hereto, we combined chromatin immunoprecipitation (ChIP) coupled with *S.* Typhimurium whole-genome tiling arrays (ChIP-chip) and transcriptomics comparing gene expression in a *fabR* deletion mutant and wildtype *S.* Typhimurium SL1344. ChIP-chip facilitates the identification of direct regulatory targets on a genome-wide scale *in vivo* [32] and does not rely on *in vitro* observations (as most biochemical methods) or the sometimes spurious presence of consensus DNA-binding sites (as in *in silico* motif detection algorithms). Combining it with a transcriptomics approach allows the discrimination between direct and indirect target genes and reduces its inherent noise [33–36] This provided the first evidence for the direct repression of *fabA*, *fabB* and *yqfA* expression by FabR in *S.* Typhimurium, confirming current knowledge generated in *E. coli*. We showed that *fabB* overexpression results in a decreased biofilm formation, indicating a role for FabB (involved in UFA biosynthesis) in mediating the effect of *fabR* on biofilm formation. Altered UFA synthesis might impact on biofilm formation in several ways, either by alterations in membrane fatty acid composition, membrane fluidity and surface properties or by a role of free UFA’s as signaling molecules. Moreover, next to UFA biosynthesis, a number of other genes, known to be involved in biofilm formation, identified to be (in)directly regulated by FabR (e.g. ribosomal genes, *ompA*, *ompC*, *ompX*, *osmB*, *osmC, sseI*), could possibly contribute to the effect of FabR on biofilm formation.

## Methods

### Bacterial strains, plasmids and media

All strains and plasmids used in this study are listed in Table 1. *Salmonella* cultures were routinely grown with aeration in Luria-Bertani (LB) broth, on LB agar plates containing 15 g/l agar (Invitrogen) or in tryptic soy broth (BD Biosciences, 30 g/l) diluted 1/20 (TSB 1/20), with the addition of antibiotics (ampicillin (Ap), 100 μg/ml; chloramphenicol (Cm), 25 μg/ml), if appropriate.

**Table 1:** Bacterial strains and plasmids

Name	Description	Reference
Strains		
*E. coli* DH5α	F^−^ ϕ80Δ*lacZM15* Δ(*lacZYAargF)*U169 *deoP recA1 endA1 hsdR17* (r_k_^−^ m_k_^−^) λ^−^	Gibco BRL
*E. coli* TOP10F’	F’ {*lacI*q Tn*10*(TetR)} *mcrA* ∆(*mrr*-*hsdRMS*-*mcrBC*) φ80*lacZ*∆M15 ∆*lacX74 deoR recA1 araD139* ∆(*ara*-*leu*)7697 *galU galK rpsL* (StrR) *endA1 nupG*	Invitrogen
*S.* Typhimurium SL1344	Wildtype strain, *xyl hisG rpsL*; virulent; Sm^R^	[82]
CMPG5624	*S.* Typhimurium SL1344, Δ*fabR*	This study
CMPG5825	*S.* Typhimurium SL1344, *fabR-M9*	This study
Plasmids		
c3390	SpeI-*myc9*-SpeI cassette in pBlueskript SKII-	[39]
pCP20	*flp*, ts-rep-[*ciI857*](λ) ts, Ap^R^, Cm^R^	[38]
pCMPG5553	*yqfA* cloned XbaI/SacI in pFAJ1708	This study
pCMPG5678	*fabR* cloned EcoRI/BamHI in pFAJ1708	This study
pCMPG10118	*fabA* cloned XbaI/EcoRI in pFAJ1708	This study
pCMPG10119	*fabB* cloned XbaI/EcoRI in pFAJ1708	This study
pFPV25	Promoter-trap vector constructed by inserting an EcoRI-HindIII fragment containing a promoterless *gfpmut3* [83] into plasmid pED350 (colE1, bla, mob) [84]; ApR	[85]
pKD3	Plasmid used as template for construction of *Salmonella* mutants; Ap^R^, Cm^R^	[38]
pKD46	Lambda Red helper plasmid, Ap^R^	[38]

Standard protocols were used for molecular cloning [37]. Cloning steps were performed using *E. coli* DH5α and TOP10F’ and the final, constructed plasmids were electroporated to the *S.* Typhimurium SL1344 strains using a Bio-Rad gene pulser. Restriction enzymes were purchased from New England Biolabs and used according to the manufacturer’s instructions. All primers used and their purposes are listed in Additional file 1: Table S2. The sequences used for primer construction were obtained from the complete genome sequence of *S.* Typhimurium SL1344, as available via the website of the Sanger Institute (U.K.), (http://www.sanger.ac.uk/Projects/Salmonella). Plasmids pCMPG5678, pCMPG5553, pCMPG10118 and pCMPG10119 were constructed by cloning respectively the PCR-amplified *fabR* (STM4127), *yqfA* (STM3049), *fabA* (STM1067) and *fabB* (STM2378) coding sequences, as EcoRI/BamHI (*fabR*), XbaI/SacI (*yqfA*) or XbaI/EcoRI (*fabA* and *fabB*) fragments, downstream of the constitutive *nptII* promoter into the RK2 based plasmid pFAJ1708. The *S.* Typhimurium SL1344 ∆*fabR* (CMPG5624) mutant was constructed using the procedure described by Datsenko and Wanner [38], starting from plasmid pKD3. A strain with a chromosomally encoded 9xMyc epitope-tagged FabR was constructed as previously described [35], using primers PRO379, PRO254, PRO494, PRO495, pCRII TOPO (Invitrogen), c3390 [39]. All strains and constructs were finally verified by PCR and sequencing analysis.

### Phenotypic assays

Two different biofilm assays were used: (i) biofilms were formed using the static high-throughput peg system and at the bottom of petri dishes at 16 or 25 °C in TSB 1/20 for 48 h, as previously described [40] . The only modification being that 150 instead of 200 μl was added to each well of the microtiter plate. For biofilm formation studies at 30 °C, the high-throughput peg system was incubated in a humid environment to minimize evaporation from the wells. To test the effect of free fatty acids on biofilm formation, two-fold serial dilutions of the fatty acids were prepared in the peg system as described previously [41]. Results of the peg-based assays are shown in the figures as a percentage of biofilm formed, compared to wildtype *S.* Typhimurium strain SL1344 (100 %) and error bars represent the standard deviation of at least three independent measurements. (ii) Biofilm formation on the bottom of small polystyrene petri dishes (60 mm diameter, Greiner Bio-One) was performed by adding 10 ml of a 1:100 dilution of the particular S. Typhimurium strain into TSB 1/20 broth. After 48 h stationary incubation at 25 °C, the bacteria formed a biofilm layer at the bottom. Biofilms cells were harvested by scraping off the biofilm.

Growth curves of wildtype and mutant strains were recorded using a Bioscreen C system (Oy Growth Curves Ab Ltd). Overnight cultures of the strains were 1:100 diluted into LB and TSB 1/20 broth into three separate wells of a 100 well honeycomb plate (three biological repeats) and grown at 25 °C for 48 h. The experiments were performed under continuous shaking conditions and the optical density (OD_595_), reflecting the bacterial growth, was measured every 15 min.

As an additional phenotypic biofilm-related assay we performed: Congo red (CR) morphotype formation tests. LB agar without NaCl supplemented with Congo red (40 μg/ml) and Coomassie brilliant blue (20 μg/ml) (CR agar plates), incubated at room temperature (*i.e.* 25 °C), was used to judge colony morphology and color (*i.e.* rdar morphotype formation), reflecting proper EPS production (mainly curli and cellulose for *S.* Typhimurium) [10].

### ChIP-chip analysis

*Salmonella* strains were cultured under free-living TSB conditions (TSB 1/20, 200 rpm, 25 °C) until an OD_595_ of 0.3 (ca. 2 x 10^8^ cells) was reached (ca. 6 h). ChIP experiments were performed as previously described [35] on the *fabR-M9 S.* Typhimurium SL1344 strain (CMPG5825). ChIP-enriched DNA fragments were blunted and amplified via ligation-mediated (LM) PCR using PRO336 and PRO337 [42], and sent to NimbleGen Systems, Inc. ChIP samples were labelled with Cy5 and hybridized against a genomic DNA reference, labelled with Cy3, on *S*. Typhimurium LT2 whole genome tiling arrays. The arrays consisted of 387,000 unique 50-mer probes covering the whole *S*. Typhimurium LT2 genome and pSLT plasmid with a moving window overlap of 12 bases [43]. Note that *S.* Typhimurium SL1344 does not contain the prophages Fels-1 and Fels-2 as compared to *S.* Typhimurium LT2 [44, 45]. Raw data were normalized by polynomial regression and log_2_ ratios of ChIP over reference (log_2_ enrichment ratios) were calculated (Matlab). To identify FabR-bound enriched regions in the ChIP-chip data, peak detection was performed with Mpeak [46] using the log_2_ enrichment ratios as input and performing first ‘simple cluster detection’ and then ‘peak detection’. All default parameter values were used, except for the ‘minimum number of probes’, which was set to 45 to account for the sonication process. Sonication produces DNA fragments of 800 bp on average, such that the ensemble of all retrieved FabR-bound DNA fragments for a specific target gene span a region of approximately 1700 bp, corresponding to ca. 45 probes in our tiling array design. The default Mpeak *p*-value is 0.01. Log_2_ enrichment ratios and identified peaks were visualized with SignalMap software (NimbleGen Systems, Inc.).

### Regulatory motif detection

The promoter or intergenic regions of the confirmed FabR-bound target genes were screened for conserved sequences using MotifSampler [47]. The promoter regions were extracted from the *S.* Typhimurium SL1344 genome sequence, as available via the website of the Sanger Institute (U.K.) (http://www.sanger.ac.uk/Projects/Salmonella). The whole promoter (*fabA*, *fabB* and *yqfA*) or intergenic (*hpaR*/*hpaG* and *ddg*/*yfdZ*) regions were used as seeds for the algorithm, with default parameters and motif widths ranging between 8 and 15 bp.

### ChIP-qPCR analysis

ChIP was repeated for two biological replicates of both *fabR-M9* (CMPG5825, ChIP samples) and wildtype cultures (mock ChIP samples) grown under free-living TSB conditions (TSB 1/20, 200 rpm, 25 °C, 6 h) as described above, and the enrichment of the promoter regions was assessed by qPCR with *dnaG* [48] as endogenous control. The enrichment ratios of ChIP over mock ChIP samples were calculated as RQ = 2^-(∆Ct ChIP -∆Ct mock ChIP)^, in which ∆Ct_ChIP_ is Ct_gene test_ – Ct_*dnaG*_ for the ChIP samples and ∆Ct_mock ChIP_ is Ct_gene test_ – Ct_*dnaG*_ for the mock ChIP samples. Additionally, cells were cultured under LB conditions (LB, 200 rpm, 25 °C) until an OD_595_ of 0.7 (ca. 5 x 10^8^ cells) was reached (ca. 5-6 h)) and biofilm conditions (TSB 1/20, 25 °C, 48 h at the bottom of petri dishes as described above). qPCR reactions were performed on 1 μl of ChIP and mock ChIP-enriched DNA and serial dilutions of input DNA on the StepOnePlus (ABI) using PowerSYBR Green PCR Master Mix (Applied Biosystems), according to the manufacturer’s instructions. ChIP-qPCR primers (Additional file 1: Table S2) were designed with Primer Express 3.0 (Applied Biosystems). All reactions were at least performed in triplicate. Amounts of PCR product in the ChIP and mock ChIP samples were determined using the StepOne™ (version 2.1) and DataAssist™ (version 2.0) software from Applied Biosystems.

### Transcriptome microarray analysis and qRT-PCR validation

*S.* Typhimurium SL1344 wildtype and CMPG5624 (∆*fabR*) were cultured as discussed above under free-living TSB conditions. Samples were treated with 1/5 volume ice-cold phenol:ethanol mixture (5:95) and transferred to a microcentrifuge tube which was immediately frozen in liquid nitrogen and stored at −80 °C. Total RNA was isolated with the Qiagen RNeasy mini kit according to the manufacturer’s protocol. Contaminating genomic DNA was removed from the RNA samples with Turbo DNA-free (Ambion). Removal of DNA was checked by PCR. Prior to labeling, the concentration of total RNA was determined by measuring the A_260_ with a NanoDrop spectrophotometer (ND-1000). RNA was labeled with Cy5 and Cy3 by reverse transcription [9]. Hybridizations were performed in color flip on *S.* Typhimurium arrays containing 70-mer oligos representing all LT2 annotated genes (Operon), spotted in duplicate on CodeLink Activated slides (Amersham Biosciences), as previously reported [35]. Data were Loess normalized with the LIMMA BioConductor package without performing a background correction. Differentially expressed genes were detected by *t*-test with multiple testing correction (*p*-value < 0.02 and absolute fold change > 1.3).

Expression of a selected number of genes was additionally assayed through qRT-PCR (two biological repeats), as previously discussed [40]. The used qRT-PCR primers are listed in Additional file 1: Table S2. cDNA was prepared starting from 100 ng of DNase-treated RNA extracted from cells cultured under the conditions as specified above. Normalization of the target gene’s expression was performed using *dnaG*, *gyrB*, *recA*, *rfaH* and *rrsG* as endogenous controls using the DataAssist™ (version 2.0) software package from Applied Biosystems.

## Results

### *FabR involvement in* S. *Typhimurium biofilm formation*

**Fig. 1:**
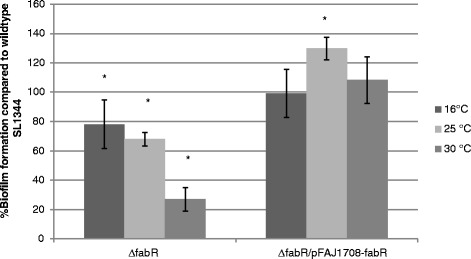
Biofilm formation by an isogenic *fabR* mutant. The level of biofilm formation at the indicated temperatures is expressed as a percentage of wildtype SL1344 biofilm formation at the respective temperatures. The data are representative of three independent experiments (*n* = 3), with at least 4 replicates each. The error bars denote standard deviations between the independent experiments. For each temperature a one-sample *t*-test was performed to compare the mean biofilm formation of the mutant (expressed as a percentage of wildtype SL1344 biofilm formation) to 100 %. Significant changes (*p*-value <0.05) in the level of biofilm formation as compared to the wildtype at the same temperature are indicated with an asterix (*). SL1344: S*.* Typhimurium SL1344 wildtype strain; ∆*fabR*: *S.* Typhimurium SL1344 ∆*fabR* mutant (CMPG5624); ∆*fabR-*pFAJ1708-*fabR trans* complemented *S.* Typhimurium SL1344 ∆*fabR* mutant (pCMPG5678/CMPG5624)

Mutant screening revealed a *S.* Typhimurium SL1344 *fabR* deletion mutant (CMPG5624) to show an impaired biofilm formation at 16, 25 and 30 °C when grown in TSB 1/20 for 48 h. Different temperatures were tested because the regulation of biofilm formation in *Salmonella* is known to be strongly temperature dependent [4]. The observed biofilm defect could be complemented by introducing *fabR in trans* (pCMPG5678) (Fig. 1). The complementation experiment also indicated that balanced *fabR* expression -with respect to expression level and/or kinetics- is necessary for proper biofilm formation. Indeed, the complemented ∆*fabR* mutant, in which *fabR* expression is driven by a constitutive promoter, shows increased biofilm formation relative to the wildtype, especially at 25 °C. Since for the ∆*fabR* mutant no defect in planktonic growth (in TSB 1/20 nor LB) or CR morphotype formation (reflecting rdar morphotype expression) [10] was observed (data not shown), it is unlikely that *fabR* deletion affects biofilm formation through an interference with respectively its growth characteristics or EPS (cellulose or curli) production.

### Genome-wide identification of direct FabR target genes by ChIP-chip

ChIP-chip analysis was performed to unravel the direct, *in vivo* transcriptional target network of the *S.* Typhimurium SL1344 FabR regulator on a genome-wide scale (GEO record: GSE52877)*.* It was performed using CMPG5825, a *S.* Typhimurium SL1344 strain expressing a 9xMyc-tagged FabR protein from the native *fabR* chromosomal locus, on *S.* Typhimurium SL1344 whole genome tiling arrays, as described in Materials and Methods. The ChIP-chip experiment was performed for cells cultured for 6 h under free-living TSB conditions (TSB 1/20, 200 rpm, 25 °C). Several factors needed for initiation of biofilm formation are already present in planktonic cells prior to attachment [49]. There is thus a strong possibility that the biofilm defect of the Δ*fabR* mutant is caused by changes in these factors within planktonic cells.

In this analysis we identified 30 individual peaks reflecting putative FabR-DNA-binding sites. Peaks located within coding sequences (STM0955, STM1333, STM1334.c, STM1336, STM2108, STM2249, STM2250, STM2419, STM2617, STM3281, STM3814, and STM3815) were for the time being discarded from further analysis. After merging individual peaks in the same genomic regions, our analysis identified twelve possible FabR targets (Table 2). These twelve putative FabR targets (*i.e.* intergenic or promoter regions corresponding to one or two gene(s), respectively), are involved in different processes, as indicated in Table 2. Surprisingly, FabR appears to bind in the promoter regions of three copies of the *S.* Typhimurium initiator tRNA genes that encode tRNA^fMet^ [50], according to the predicted FabR binding peaks. The putative FabR-DNA-binding sites situated in the promoter regions of ribosomal genes and tRNAs were, however, not the focus of our further investigation. Of the five remaining putative targets, two are known FabR targets in *E. coli* [29, 51, 52]: *fabB*, encoding β-ketoacyl-ACP synthase catalysing the rate-limiting and essential reaction during the unsaturated fatty acid biosynthesis [53], and *yqfA*, encoding an inner membrane protein of the hemolysin 3 family with putative oxidoreductase function [54], while the three others are for the first time linked to *in vivo* FabR binding. These novel FabR target sites include (i) the intergenic region between the divergently transcribed *hpaR* and *hpaG* genes, both involved in aromatic catabolism (4- and 3-hydroxyphenylacetate degradation) [55, 56]; (ii) the intergenic region between divergently transcribed *metY* and *argG* encoding a tRNA and argininosuccinate synthase, respectively; and (iii) the intergenic region between the convergently transcribed genes *ddg*, involved in lipid A biosynthesis, and *yfdZ*, encoding an aminotransferase. The latter genomic organization makes it, however, unlikely for FabR to exert any regulatory function in this region.

**Table 2:** Putative FabR targets identified using ChIP-chip

Gene ID^a^	Name^a^	Possible FabR Target^b^	Function^a^	Functional Class^b^
STM1100	*hpaR* (<)	IR *hpaR*-*hpaG*	4-Hydroxyphenylacetate catabolism	1
STM1101	*hpaG* (>)		4-Hydroxyphenylacetate catabolism	1
STM1336	*rplT* (>)	P *pheS*	50S ribosomal protein L20	2
STM1337	*pheS* (>)		Phenylalanyl-tRNA synthetase subunit alpha	3
STM2378	*fabB* (<)	IR *fabB*-STM2379	3-Oxoacyl-(acyl carrier protein) synthase I	4
STM2379	STM2379 (>)		5-Methylaminomethyl-2-thiouridine methyltransferase	5
STM2401	*ddg* (>)	DR *ddg-yfdZ*	Lipid A biosynthesis palmitoleoyl acyltransferase	6
STM2402	*yfdZ* (<)		Aminotransferase	7
STM2415	*gltX* (<)	IR *gltX*-*valU*	Glutamyl-tRNA synthetase	3
STM2416	*valU* (>)		tRNA	3
STM2615	STM2615 (<)	P STM2615	tRNA	3
STM2616	STM2616 (<)		Antirepressor-like protein	8
STM2616	STM2616 (<)	P STM2616	Antirepressor-like protein	8
STM2617	STM2617 (<)		Antiterminator-like protein	8
STM2989	*metZ* (>)	P *metW*	tRNA	3
STM2990	*metW* (>)		tRNA	3
STM3049	*yqfA* (<)	P *yqfA*	Putative hemolysin	6
STM3050	*yqfB* (<)		Hypothetical protein	5
STM3289	*metY* (<)	IR *metY*-*argG*	tRNA	3
STM3290.S	*argG* (>)		Argininosuccinate synthase	7
STM4148	*nusG* (>)	P *rplK*	Transcription antitermination protein	8
STM4149	*rplK* (>)		50S ribosomal protein L11	2
STM4150	*rplA* (>)	P *rplJ*	50S ribosomal protein L1	2
STM4151	*rplJ* (>)		50S ribosomal protein L10	2

^a^STM numbers, gene names, genomic orientation (< and > indicating minus and positive strand, respectively) and gene functions are taken from NCBI Refseq NC_003197 [86] and results are sorted according to their STM numbers

^b^The mentioned genes belong to the following functional classes: (1) Carbon compound degradation, (2) Ribosomal protein synthesis and modification, (3) Aminoacyl tRNA metabolism, (4) Fatty acid metabolism, (5) Conserved hypothetical protein, (6) Membrane homeostasis, (7) Amino acid biosynthesis, (8) RNA synthesis, RNA modification and DNA transcription

^b^IR indicates that both genes are possible FabR targets since the FabR binding region was situated in the intergenic region between the two mentioned divergently transcribed genes and hence contains the (putative) promoters of both genes. P points at the (putative) promoter region of the mentioned gene since the intergenic region identified during the ChIP-chip analysis was situated between two genes transcribed in the same direction and hence only contains the (putative) promoter of this gene. DR indicates that probably none of the identified genes is a putative FabR target since both adjacent genes are convergently transcribed respective to the retained intergenic FabR binding region

**Fig. 2:**
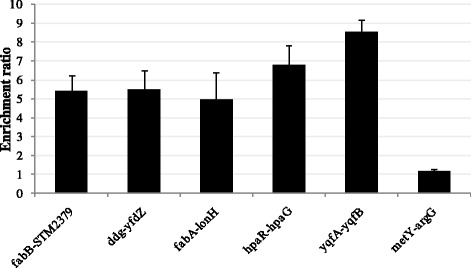
ChIP-qPCR validation of the ChIP-chip data. The validation of the ChIP-chip results by ChIP-qPCR analysis was performed as elaborated in Materials & Methods with *dnaG* as endogenous control. Values represent enrichment ratios of ChIP over mock ChIP samples, generated under free-living TSB conditions, and are averages of triplicate ChIP-qPCRs. The enrichment ratios of ChIP over mock ChIP samples were calculated as RQ = 2^-(∆Ct ChIP -∆Ct mock ChIP)^, in which ∆Ct_ChIP_ is Ct_gene test_ – Ct_*dnaG*_ for the ChIP samples and ∆Ct_mock ChIP_ is Ct_gene test_ – Ct_*dnaG*_ for the mock ChIP samples. The presented values are representative for two biological repeats and standard deviations of the three technical repeats are indicated

Further validation of the ChIP-chip results was obtained by ChIP-qPCR. As can be seen in Fig. 2, the *metY*/*argG* intergenic region appeared not to be upregulated in the ChIP-qPCR analysis, suggesting that it might represent an artifact (*i.e.* false positive) of the ChIP-chip analysis [33]. ChIP-qPCR for the verified targets was also performed on ChIP samples taken under LB and biofilm conditions. FabR appeared to bind the investigated promoter regions under these different environmental conditions as well (Additional file 1: Figure S1), substantiating the direct, *in vivo* binding of *S.* Typhimurium FabR.

Next to the two known *E. coli* targets, FabR is also known to control the expression of *fabA* in *E. coli* by binding to its promoter region [29, 52]. Although not detected on the tiling array, we could show binding of FabR to the *fabA* promoter by ChIP-qPCR, under TSB, LB and biofilm conditions. In *S.* Typhimurium, this represents the first evidence for direct *in vivo* binding of FabR to the *fabB*, *fabA* and *yqfA* promoters.

### Sequence conservation in the promoter region of direct FabR regulon members

**Fig. 3:**
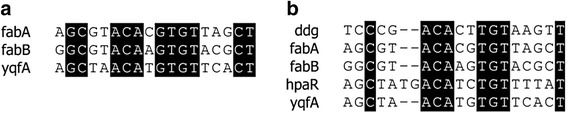
Alignment of the putative FabR binding site in the ChIP-qPCR verified *in vivo* FabR targets. **a** Alignment of the already known *E. coli* FabR targets [51] in *S.* Typhimurium SL1344; **b** Alignment of the ChIP-chip identified and ChIP-qPCR verified FabR targets in *S.* Typhimurium SL1344. All alignments were performed using MotifSampler [47]. Sequences upstream of the coding sequences of the indicated genes were taken from the complete genome sequence of *S.* Typhimurium SL1344. These input sequences comprised the full intergenic region, *i.e.* the region between the coding sequence of the FabR target gene and the upstream coding sequence, with *hpaR* and *ddg* indicating the *hpaR*/*hpaG* and *ddg*/*yfdZ* intergenic sequences, respectively. White letters with black background denote identical bases and black letters on a white background denote differing bases

The FabR targets *fabA*, *fabB* and *yqfA* were previously identified in *E. coli* starting from a common motif identified using an *in silico* phylogenetic footprinting analysis [51]. Subsequent verification of the biological significance of this motif for FabR binding indicated the importance of the sequences flanking the motif for FabR binding in the absence –but not in the presence- of unsaturated thioesters, which are the native FabR ligands [29]. The sequence alignment of the originally identified degenerative binding palindrome upstream of their coding sequences in the case of *S.* Typhimurium SL1344, is depicted in Fig. 3a. To further validate that the newly *in vivo* identified targets are controlled by FabR, their putative intergenic regions were *in silico* screened for an overrepresented motif using the motif discovery algorithm MotifSampler [47]. More specifically, a *de novo* screening procedure was performed starting from the promoter and intergenic regions of the five ChIP-qPCR validated FabR targets. As shown in Fig. 3b, essential parts of the FabR palindromic recognition sequence were retained in all of them. Given the 2 bp ‘gap’ of all other aligned sequences as compared to the *hpaR*/*hpaG* one, it seems reasonable to postulate that ACAnnTGTnnnnT constitutes the ‘core’ motif.

### Transcriptional profiling of a fabR mutant

**Fig. 4:**
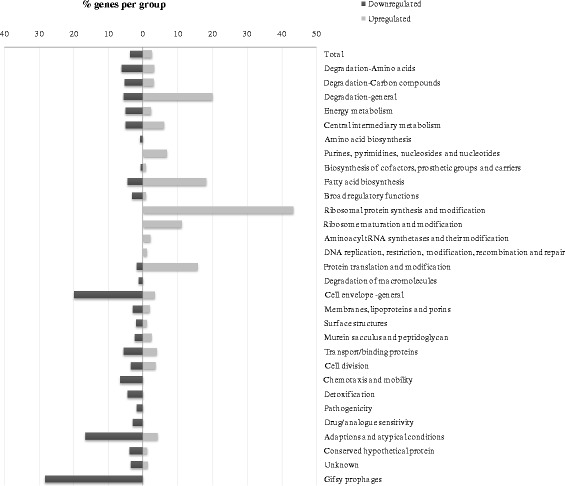
Functional classification of the differentially expressed genes in the isogenic *fabR* deletion mutant as compared to the wildtype under free-living TSB conditions. The bars represent the percentage of genes belonging to each group that were altered for absolute expression > 1.3 fold with a *p*-value < 0.02. The functional classes defined by the Welcome Trust Sanger Institute were used for this classification and the numbers behind each class represent the number of genes in this class

**Fig. 5:**
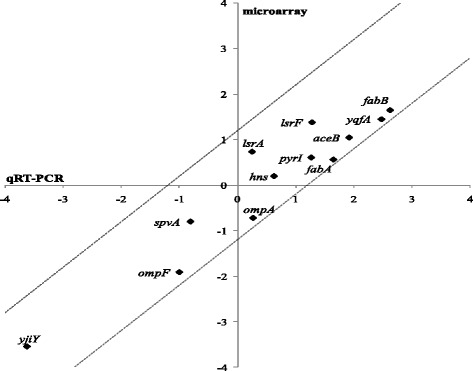
Comparison between the microarray and qRT-PCR data. The expression of a number of genes was determined using qRT-PCR for the *S.* Typhimurium SL1344 wildtype and SL1344 ∆*fabR* mutant under free-living TSB conditions. The log_2_-transformed mean value of at least three qRT-PCR technical repeats (representative for each of the two assayed biological replicates) for each gene was plotted on the X-axis and compared to the respective log_2_-transformed microarray fold change (Y-axis). All depicted qRT-PCR tested genes had a *p*-value < 0.02 under their respective microarray conditions and their qRT-PCR primers are listed in Additional file 1: Table S2. The dotted lines represent arbitral boundaries (y = x + 1.2 and y = x – 1.2) between which the corresponding qRT-PCR and microarray results show good correspondence (*i.e.* not more than a 1.2 fold divergence on log_2_ scale) with y = x being the ideal situation

Since ChIP-chip data give information on the location of a regulator (*i.e.* direct binding), not on its functioning (*i.e.* not on the downstream effects this binding causes) and since promoter regions are often enriched by ChIP-chip without being regulated by the transcription factor [33, 34, 57, 58], we complemented the ChIP-chip data with a transcriptomics study. Hereto, mRNA levels in the wildtype and the ∆*fabR* mutant (CMPG5624) were compared at the same time point and under the same experimental conditions as used for the ChIP-chip experiment (GEO record: GSE52880). In view of a stringent selection, genes with an absolute fold change > 1.3 and a *p*-value < 0.02 were considered as significant. This threshold is acceptable since most regulatory responses in nature appear to function using low level changes as a kind of energy saving solution [59]. Of all genes on the array, respectively 179 genes (3.79 %) and 119 genes (2.52 %) were significantly down- and upregulated, in the *fabR* mutant as compared to the wildtype (functionally visualized in Fig. 4). A set of 12 of these differentially expressed genes was verified by qRT-PCR measurements and as can be seen in Fig. 5, the array data are overall in good agreement with the qRT-PCR data.

The genes upregulated in the *fabR* deletion mutant were found to be strongly overrepresented in the groups of genes involved in general degradation processes (such as the fatty acid degradation genes *fadA*, *fadB* and *fadE*), fatty acid biosynthesis, biosynthesis/modification of ribosomal proteins and protein translation/modification, consistent with the putative binding sites identified during the ChIP-chip analysis. As expected, the direct targets (*fabB*, *fabA* and *yqfA*) were significantly upregulated, confirming the direct repressor function of FabR on these genes in *S.* Typhimurium (Table 3 and Additional file 1: Table S1). Moreover, combination of the transcriptomic and the ChIP-chip results provides the first experimental evidence that FabR directly binds to the *yqfA* promoter and regulates its expression. This result contrasts with recent findings by Feng and Cronan, who observed that FabR binding to *yqfA* was without physiological consequence in *E. coli* background (*i.e.* no regulatory role for FabR in regulating *yqfA* expression under their tested conditions) [29]. The data presented in Table 3 also indicate that FabR is more dedicated towards *fabB* regulation as compared to *fabA* [29, 31]. The differential expression of other genes involved in the fatty acid metabolism (Table 3) might compensate for maintaining normal cellular fatty acid composition and energy homeostasis in the *fabR* mutant. Similar crosstalk between different components of the fatty acid metabolism (biosynthesis, degradation and uptake) in fatty acid regulatory null mutants has previously been described [31]. In this context, it was also not surprising to encounter an upregulated expression of the *aceB*-*aceA*-*aceK* operon in the *fabR* deletion mutant (Table 3). This operon encodes enzymes in the glyoxylate shunt and as such connects the TCA cycle and the fatty acid metabolism, both crucial processes in balancing cellular energy levels [60]. In addition, the whole *lsrR* regulon [36] involved in AI-2 uptake and processing, was found to be significantly upregulated in a *fabR* mutant. Although still under investigation in *S.* Typhimurium [61–64], the LuxS synthesized AI-2 has previously been linked to biofilm formation [65]. The exact reason why these genes are upregulated in a *fabR* mutant, however, remains to be elucidated since no direct binding to the *lsrR* promoter region was identified in the Chip-chip (and ChIP-qPCR, data not shown) analysis. The upregulation of the genes involved in ribosomal protein synthesis and modification (Table 3 and Fig. 4) is consistent with the putative binding of FabR to such genomic regions (Table 2). Although the ChIP-chip and ChIP-qPCR experiments indicated FabR to bind to the *hpaR*-*hpaG* intergenic region, none of these genes were significantly upregulated in the *fabR* mutant.

The downregulated genes (Fig. 4, Additional file 1: Table S1) were found to be overrepresented in the classes of genes involved in cell-envelope structure (including the outer membrane protein-encoding genes *spvA*, *nmpC* (*ompD*), *ompA*, *ompC*, *ompF* and *ompX*) and adaptation processes (such as *yjiY* encoding a carbon starvation protein and the *osmB* and *osmC* genes encoding osmotically inducible proteins), as well as lysogenic Gifsy prophage genes (encoding putative virulence factors [44, 45]) (Fig. 4). This activating effect of FabR most likely happens indirectly because none of the *in vivo* ChIP-chip identified target genes showed a downregulated expression in the *fabR* mutant relative to the wildtype strain.

**Table 3:** Top-35 upregulated genes under free-living TSB conditions in the ∆*fabR* mutant versus the wildtype

Ranking^a^	ID^b^	Name^b^	Function^b^	Fold^a^	Process^b^
1	STM2378	*fabB*	3-Oxoacyl-[acyl-carrier-protein] synthase I	3.14	Fatty acid metabolism
6	STM3982	*fadA*	β-subunit of the fatty acid-oxidizing multi-enzyme complex	2.26	
8	STM3983	*fadB*	α-subunit of the fatty acid-oxidizing multi-enzyme complex	2.16	
19	STM0309	*yafH/fadE*	Acyl-CoA dehydrogenase	1.71	
34	STM0454	*ybaW/fadM*	Long-chain acyl-CoA thioesterase III	1.49	
35	STM1067	*fabA*	D-3-hydroxydecanoyl-(acyl carrier-protein)	1.48	
2	STM3049	*yqfA*	Putative hemolysin	2.73	Membrane-related
31	STM1254		Putative lipoprotein	1.51	
3	STM4078	*yneB/lsrF*	Putative fructose 1-6-phosphate aldolase	2.60	AI-2 metabolism
5	STM4079	*yneC/lsrG*	Isomerase for processing of phospho-AI-2	2.49	
7	STM4080	*lsrE*	Putative ribulose-5-phosphate 3-epimerase	2.24	
10	STM4077	*yneA/lsrB*	ABC transport protein, solute-binding component	2.13	
12	STM4071		Hypothetical protein	2.02	
13	STM4072	*ydeV/lsrK*	Sugar kinase	1.87	
17	STM4076	*ydeZ/lsrD*	ABC transporter, membrane component	1.75	
21	STM4074	*ego/lsrA*	ABC transporter ATP-binding protein	1.66	
23	STM4075	*ydeY/lsrC*	ABC transporter permease protein	1.64	
4	STM4184	*aceA*	Isocitrate lyase	2.50	Glyoxylate metabolism
11	STM4183	*aceB*	Malate synthase A	2.07	
14	STM4185	*aceK*	Isocitrate dehydrogenase kinase/phosphatase	1.86	
9	STM3195	*ribB*	3,4-Dihydroxy-2-butanone 4-phosphate synthase	2.14	Riboflavin metabolism
15	STM2935	*cysD*	ATP sulphurylase (ATP:sulphate adenyltransferase)	1.79	Sulphur metabolism
16	STM3304	*rplU*	50S ribosomal subunit protein L21	1.78	Ribosomal protein synthesis and modification
22	STM3303	*rpmA*	50S ribosomal subunit protein L27	1.65	
26	STM3209	*rpsU*	30S ribosomal subunit protein S21	1.62	
27	STM3345	*rplM*	50S ribosomal subunit protein L13	1.61	
30	STM3429	*rplX*	50S ribosomal subunit protein L24	1.51	
18	STM2970	*sdaC*	Putative serine transporter	1.72	Transport processes
20	STM1530		Putative outer membrane protein (truncation)	1.68	
28	STM2444	*cysP*	Thiosulphate-binding protein precursor	1.55	
32	STM1473	*ompN*	Outer membrane protein	1.51	
33	STM1452	*ydgR*	Putative proton/oligopeptide symporter	1.49	
24	STM0307		Putative secreted protein	1.64	Pathogenicity
25	STM0447	*tig*	Trigger factor	1.63	Protein folding
29	STM4459	*pyrI*	Aspartate carbamoyltransferase regulatory subunit	1.52	Pyrimidine metabolism

^a^Rank (#) depends on the fold change with 1 being the gene with the greatest fold induction and 35 the one with the 35^th^ fold induction. Fold change represents the differential expression of the gene in the isogenic *fabR* deletion mutant (CMPG5624) according to the wildtype under free-living TSB conditions, as detected by *t*-test with multiple testing correction. ^b^ STM numbers, gene names and gene functions are taken from the fully annotated *S.* Typhimurium LT2 genome (NCBI Refseq NC_003197) [86] and adapted according to recent literature (e.g. functions of the *lsr* genes were taken from [36, 87]). Results are sorted according to the functional classes (process) they belong to and with descending fold change in each class

### fabB is important for Salmonella biofilm formation

**Fig. 6:**
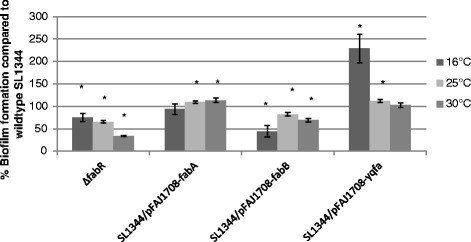
Effect on biofilm formation of individually overexpressing *fabA*, *fabB* and *yqfA*. The level of biofilm formation at the indicated temperatures is expressed as a percentage of wildtype SL1344 biofilm formation. The data are representative of three independent experiments (*n* = 3), with at least 4 replicates each. The error bars denote standard deviations between the independent experiments. For each temperature a one-sample *t*-test was performed to compare the mean biofilm formation of the different strains (expressed as a percentage of wildtype SL1344 biofilm formation) to the wildtype level of 100 %. Significant changes (*p*-value <0.05) in the level of biofilm formation as compared to the wild type at the same temperature are indicated with an asterix (*)SL1344: *S.* Typhimurium SL1344 wildtype strain; ∆*fabR*: *S.* Typhimurium SL1344 ∆*fabR* mutant (CMPG5624); SL1344/pFAJ1708-*fabA*: *S.* Typhimurium SL1344 overexpressing *fabA* (pCMPG10118/SL1344); SL1344/pFAJ1708-*fabB*: *S.* Typhimurium SL1344 overexpressing *fabB* (pCMPG10119/SL1344); SL1344/pFAJ1708-*yqfA*: *S.* Typhimurium SL1344 overexpressing *yqfA (*pCMP5553/SL1344)

Finally, we investigated whether the biofilm defect of a *fabR* mutant could be attributed to the observed enhanced expression of any of the direct targets *fabA*, *fabB* or *yqfA*. Hereto we individually overexpressed these genes by respectively introducing plasmids pCMPG10118, pCMPG10119 and pCMPG5553 in the wildtype strain. In these plasmids the genes are cloned downstream of the constitutive *nptII* promoter. As indicated in Fig. 6, overexpression of *fabA* and *yqfA* does not result in a reduced biofilm formation. At 16 °C *yqfA* overexpression even strongly increases biofilm formation, a phenotype which could prove interesting for further investigation. Overexpression of *fabB,* however, does result in a decreased biofilm formation, indicating a role for *fabB* in mediating the effect of *fabR* on biofilm formation. At 25 and 30 °C, the biofilm defect is less pronounced in the *fabB* overexpressing strain than in the *fabR* mutant, suggesting that next to *fabB* also other *fabR* targets might play a role in biofilm formation. At 16 °C, however, the biofilm defect is much more pronounced in the *fabB* overexpressing strain than in the *fabR* mutant. This could possibly be explained by the simultaneous, counteracting effects in the *fabR* mutant of *fabB* upregulation (reduced biofilm) and *yqfA* upregulation (increased biofilm).

FabB plays a key role in UFA synthesis by catalyzing the elongation of the cis-3-decenoyl-ACP produced by FabA. Consistently, studies in *E. coli* indicated that *fabB* overexpression strongly increases UFA production [29, 66]. Given the structural similarity of these UFA’s with the DSF (diffusible signaling factor) family of compounds, which is known to induce biofilm dispersion [67], we hypothesized that the biofilm defect of the FabB overexpressing strain could be mediated by increased levels of free UFA’s, acting as biofilm dispersing molecules. Adding a concentration range of different UFA’s (cis-5-dodecenoic acid, cis-9-hexadecenoic acid and cis-11-hexadecenoic acid) to the growing biofilm, however, did not affect the amount of biofilm formed (data not shown).

## Discussion

In this study, we showed that the unsaturated fatty acid biosynthesis regulator FabR is involved in *S.* Typhimurium biofilm formation. To unravel how this regulator impinges on *Salmonella* biofilm formation, a combinatorial high-throughput approach, combining ChIP-chip with transcriptomics, was applied. High-throughput ChIP-chip analysis allowed the identification of *in vivo* FabR binding sites reflecting potential target genes (Table 2). Firstly, all the previously identified *E. coli* FabR transcriptional target genes (*fabA*, *fabB* and *yqfa*) were shown to be direct *S.* Typhimurium FabR targets as well, validating our approach. Direct binding to the promoter region of *fabA*, however, was only observed using ChIP-qPCR. The failure to detect FabR binding to this site by the ChIP-chip technique could be due to the sensitivity of the tiling array and/or stringency during the hybridization process [68] or the failure to randomly amplify this specific genomic region [69, 70]. Secondly, ChIP-chip also identified some new, direct FabR targets, *i.e.* the intergenic regions between *hpaR*/*hpaG* and *ddg*/*ydfZ*. The latter two genes (*ddg* and *yfdZ*) are, in contrast to *hpaR* and *hpaG*, convergently transcribed, making it unlikely for FabR to exert any direct regulatory function on their expression. Thirdly, our ChIP-chip analysis also identified a number of yet putative FabR target genes (e.g. genes involved in tRNA metabolism, ribosome synthesis and translation). These might also represent real *in vivo* FabR targets. Together, these data indicate that FabR only has a very limited regulon under the tested conditions. This contrasts to the broad regulon of global transcriptional regulators such as H-NS (ca. 745 direct target genes) [43] or FNR (ca. 100 direct target genes) [71] and the larger regulon of more specific regulators such as the invasion regulator HilA (ca. 20 direct target genes) [35].

Combining the ChIP-chip results with transcriptomics data provided information on the biological relevance of FabR binding for the identified regions. This combinatorial approach provides the first experimental evidence that FabR directly binds to and regulates the expression of *yqfA*, whereas for *E. coli* only evidence for binding has been given [29]. Moreover, we were able to extrapolate the previously observed higher tendency of FabR towards *fabB* regulation as compared to *fabA*, from *E. coli* to *S.* Typhimurium [29, 31]. Although the ChIP-chip and ChIP-qPCR experiments indicated FabR to bind to the *hpaR*-*hpaG* intergenic region, none of these genes were significantly upregulated in the *fabR* mutant. Several possible explanations have been reported before for this observed lack of correlation between ChIP-chip and transcriptomics results (e.g. [57, 58]). Some of these might also explain transcription factor binding to the intergenic region between convergently transcribed genes (such as FabR binding between *ddg*/*ydfZ*). In brief, the transcription factors might play other roles than regulating transcription or only have a minor impact on transcription levels. The binding sites may either serve as storage sites buffering the free pool of regulators, or have no physiological role under the given conditions, and/or depend on the presence or absence of other factors. Indeed, occupancy of a promoter region by a transcriptional regulator can be a necessary but not a sufficient condition for its transcriptional activity. In this respect, it was shown that FabR binding to the well-known *fabA* and *fabB* targets does not necessarily require unsaturated thioester ligands, but is enhanced in their presence [29, 52]. An additional substantiation that the newly identified FabR targets are not just false positives was generated using *in silico* motif detection. Indeed, the *hpaR*/*hpaG* and *ddg*/*yfdZ* intergenic regions shared a common consensus motif with the previously identified FabR targets. The degeneracy of the retrieved motif (and the above mentioned ‘gap’), however, probably limit(s) the use of *in silico* prediction algorithms based on sequence data alone to map the FabR regulon.

As we found a *fabB* overexpressing strain to partly mimic the biofilm defect of the *fabR* mutant, the effect of FabR on biofilms can be attributed at least partly to the observed enhanced expression of its direct target *fabB*. FabB plays a key role in UFA synthesis by catalyzing the elongation of the cis-3-decenoyl-ACP produced by FabA. In *E. coli* FabB overproduction has been shown to increase the synthesis of UFA’s and to enhance the UFA contents of membrane phospholipids [29, 66]. The observation that a *fabA* overexpressing strain does not show this biofilm defect, although FabA and FabB catalyze two subsequent steps in the same pathway, can be explained by the assumption that FabB catalyses the rate limiting step. Indeed, in *E. coli* FabA overproduction was shown to increase the levels of SFA moieties rather than the levels of UFA’s, an effect that was found to be nullified when both FabA and FabB were overproduced. This indicates that FabB is the limiting step in UFA synthesis and any excess cis-3-decenoyl-ACP produced by FabA would be diverted to the saturated fatty acid synthetic pathway [72]. Different, yet elusive, links between UFA synthesis and biofilm formation can be inferred. Firstly, the alterations in membrane fatty acid composition potentially impact on surface properties (roughness, cell surface charge, hydrophobicity, exposure of certain proteins, *etc.*) and biofilm formation. Membrane fluidity was indeed demonstrated to be essential in controlling swarming, a multicellular behaviour related to biofilm formation [73], and a biofilm phenotype-specific shift in membrane fatty acid composition has already been reported for *S.* Enteritidis [74]. Furthermore, fatty acids were also encountered in the EPS fraction of rdar-expressing *S.* Enteritidis strains [75]. Secondly, energy homeostasis, partly dependent on cellular fatty acid metabolism, has also been correlated with the energy-consuming *Salmonella* biofilm formation process [76]. Consistent with this, we not only noticed differential regulation of fatty acid-related genes, but also an alteration of the glyoxylate metabolism. Thirdly, as the UFA’s synthesized by FabA and FabB show high similarities with DSFs (diffusible signaling factors), a known class of biofilm dispersing compounds [67], an alternative potential mechanism through which FabR could impact on biofilm formation is by increasing the levels of free UFA’s acting as biofilm dispersing molecules. However, as we found that exogenous addition of different UFA’s did not affect biofilm formation, this role of FabR in biofilm signaling is unlikely.

Next to UFA biosynthesis, a number of other processes regulated by FabR could possibly contribute to the effect of FabR on biofilm formation. ChIP-chip and microarray analysis indicated a direct FabR binding to and upregulation of ribosomal genes. The finding of Boehm *et al*. that ribosomal stress induces *E. coli* biofilm formation suggests a possible role for ribosome overexpression in biofilm reduction [77]. Also, direct links between *Salmonella* biofilm formation and genes downregulated in a *fabR* deletion mutant, such as *ompA* [64], *ompC* [78], *ompX* [79], *osmB* [80], *osmC* [79], *sseI* [78], have previously been identified, making them potential targets through which FabR could act on biofilm formation. Several of these repressed genes encode outer membrane proteins (*ompA*, *ompC*, *ompX, osmB*). *Salmonella* mutants in *ompA* and *ompC* have been shown to be deficient in biofilm formation on polystyrene and cholesterol-coated surfaces respectively [64, 78], whereas the expression of *ompX* and *osmC* has been shown to be activated within *Salmonella* biofilms [79]. These outer membrane proteins are important for biofilm formation possibly because they mediate electrostatic interactions between salmonellae and the surface, promote overall biofilm health e.g. as nutrient channels, or have regulatory functions within biofilms [81].

## Conclusions

In conclusion, we have shown that FabR is involved in *Salmonella* biofilm formation. In addition, we have illustrated that *S.* Typhimurium FabR has a limited regulon by combining ChIP-chip analysis with dedicated expression analysis. It directly controls the expression of *fabA*, *fabB* and *yqfA* by direct binding to their promoter regions. This confirms current knowledge generated in *E. coli*, but is the first evidence for the direct regulation of these genes by FabR in *S.* Typhimurium. Moreover, novel direct FabR targets were identified. FabB overexpression was shown to partly mimic the biofilm defect of the *fabR* mutant, indicating that the effect of FabR on biofilm formation can be attributed at least partly to its effect on *fabB* expression. Exploitation of the expression analysis data, allowed us to put forward some additional putative targets (direct and indirect) through which FabR might impact on biofilm formation. Overall, our results point at the importance of FabR and UFA biosynthesis in *Salmonella* biofilm formation and their role as potential targets for biofilm inhibitory strategies.

## Additional file

Additional file 1: Supplementary material. (DOCX 66 kb)
