# Supplementary material for: FabR regulates Salmonella biofilm formation via its direct target FabB
Source: BMC Genomics. 2016 Mar 22;17:253. doi: 10.1186/s12864-016-2387-x (PMC4804515; doi:10.1186/s12864-016-2387-x)
Supplement: Supplementary file 1 — Supplementary material. (DOCX 66 kb) [file 12864_2016_2387_MOESM1_ESM.docx]

**Figure S2. ChIP-qPCR validation of the ChIP-chip data under Biofilm and LB conditions**

1. Biofilm conditions
2. LB conditions

The validation of the ChIP-chip results by ChIP-qPCR analysis was performed as elaborated in Materials & Methods with *dnaG* as endogenous control. Values represent enrichment ratios of ChIP over mock ChIP samples, generated under Biofilm (A) and LB (B) conditions, and are averages of triplicate ChIP-qPCRs. The enrichment ratios of ChIP over mock ChIP samples were calculated as RQ = 2^-(∆Ct ChIP -∆Ct mock ChIP)^, in which ∆Ct_ChIP_ is Ct_gene test_ – Ct*_dnaG_* for the ChIP samples and ∆Ct_mock ChIP_ is Ct_gene test_ – Ct*_dnaG_* for the mock ChIP samples. The presented values are representative for two biological repeats and standard deviations of the three technical repeats are indicated.

Table S1: Microarray results under free-living TSB conditions: list of genes significantly (p < 0.02) up (> 1.3 fold) - and downregulated (< -1.3 fold) in ∆*fabR* mutant as compared to wildtype SL1344.

| **Gene name** | **GenBank ID as indicated in microarray data** | **STY^a^ replaced by STM^b^ closest homologue (if present)** | **Fold change** | **p-value** |
| --- | --- | --- | --- | --- |
| *fabB* | STM2378 | STM2378 | 3.135 | 2.05E-05 |
| *yqfA* | STY3205 | STM3049 | 2.730 | 1.31E-03 |
| *yneB* | STY3792 | STM4078 | 2.602 | 1.25E-04 |
| *aceA* | STM4184 | STM4184 | 2.503 | 5.47E-06 |
| *yneC* | STM4079 | STM4079 | 2.489 | 4.36E-04 |
| *fadA* | STM3982 | STM3982 | 2.256 | 5.42E-03 |
|  | STM4080 | STM4080 | 2.240 | 1.50E-05 |
| *fadB* | STY3577 | STM3983 | 2.157 | 9.56E-04 |
| *ribB* | STM3195 | STM3195 | 2.142 | 2.43E-05 |
| *yneA* | STY3793 | STM4077 | 2.127 | 4.72E-04 |
| *aceB* | STY4401 | STM4183 | 2.067 | 6.77E-05 |
|  | STM4071 | STM4071 | 2.022 | 1.49E-04 |
| *ydeV* | STM4072 | STM4072 | 1.874 | 1.14E-04 |
| *aceK* | STM4185 | STM4185 | 1.864 | 7.96E-05 |
| *cysD* | STM2935 | STM2935 | 1.793 | 3.83E-04 |
| *rplU* | STY3483 | STM3304 | 1.781 | 5.98E-04 |
| *ydeZ* | STY3794 | STM4076 | 1.748 | 6.49E-04 |
| *sdaC* | STY3109 | STM2970 | 1.721 | 7.07E-05 |
| *yafH* | STM0309 | STM0309 | 1.705 | 2.54E-04 |
|  | STM1530 | STM1530 | 1.678 | 1.19E-04 |
| *ego* | STM4074 | STM4074 | 1.664 | 3.04E-04 |
| *rpmA* | STY3482 | STM3303 | 1.654 | 7.91E-05 |
| *ydeY* | STY3795 | STM4075 | 1.644 | 1.09E-03 |
|  | STY0352 | STM0307 | 1.639 | 1.20E-03 |
| *tig* | STY0489 | STM0447 | 1.630 | 3.08E-04 |
| *rpsU* | STY3388 | STM3209 | 1.617 | 1.75E-05 |
| *rplM* | STY3525 | STM3345 | 1.611 | 1.72E-04 |
| *cysP* | STY2681 | STM2444 | 1.545 | 2.83E-03 |
| *pyrI* | STM4459 | STM4459 | 1.524 | 2.53E-04 |
| *rplX* | STY4369 | STM3429 | 1.514 | 1.23E-04 |
|  | STY1867 | STM1254 | 1.510 | 5.17E-04 |
| *ompN* | STM1473 | STM1473 | 1.510 | 4.78E-03 |
| *ydgR* | STM1452 | STM1452 | 1.494 | 1.54E-04 |
| *ybaW* | STM0454 | STM0454 | 1.491 | 1.07E-03 |
| *fabA* | STY1088 | STM1067 | 1.477 | 6.52E-03 |
| *gudD* | STM2960 | STM2960 | 1.472 | 6.23E-04 |
| *rpsN* | STY4371 | STM3427 | 1.471 | 7.39E-04 |
| *rpmH* | STY3939A | STM3839 | 1.468 | 1.34E-02 |
| *rpsH* | STY4372 | STM3426 | 1.468 | 1.97E-05 |
| *infA* | STY0951 | STM0953 | 1.465 | 4.81E-03 |
| *cysN* | STM2934 | STM2934 | 1.452 | 6.82E-06 |
| *rplN* | STY4368 | STM3430 | 1.451 | 3.86E-03 |
| *rpmB* | STY4066 | STM3728 | 1.450 | 1.98E-04 |
| *rpsI* | STY3524 | STM3344 | 1.448 | 1.07E-03 |
| *rplV* | STY4363 | STM3435 | 1.443 | 1.20E-04 |
| *rplF* | STY4373 | STM3425 | 1.440 | 6.81E-03 |
| *rplK* | STY3736 | STM4149 | 1.432 | 1.55E-04 |
|  | STY2908 | STM2788 | 1.429 | 3.25E-03 |
| *garL* | STM3249 | STM3249 | 1.427 | 4.79E-03 |
|  | STY2907 | STM2787 | 1.427 | 5.84E-04 |
| *rpsB* | STY0239 | STM0216 | 1.426 | 7.67E-04 |
| *rpsR* | STY4749 | STM4393 | 1.424 | 1.38E-03 |
| *accB* | STY3559 | STM3379 | 1.422 | 7.29E-03 |
| *ybeC* | STM0632 | STM0632 | 1.419 | 1.32E-03 |
| *nifS* | STY2789 | STM2543 | 1.416 | 8.08E-06 |
|  | STM2786 | STM2786 | 1.414 | 1.85E-03 |
| *fkpA* | STM3453 | STM3453 | 1.411 | 5.22E-05 |
| *cspE* | STY0678 | STM0629 | 1.406 | 1.38E-02 |
| *yfhJ* | STY2783 | STM2537 | 1.405 | 5.03E-04 |
| *purB* | STY1272 | STM1232 | 1.399 | 1.42E-02 |
| *rplE* | STY4370 | STM3428 | 1.398 | 7.24E-03 |
|  | STY2905 | STM2786 | 1.398 | 1.58E-02 |
| *purF* | STY2592 | STM2362 | 1.395 | 4.24E-03 |
| *nifU* | STY2788 | STM2542 | 1.388 | 1.25E-03 |
| *yhdV* | STY3572 | STM3392 | 1.387 | 2.59E-03 |
| *gudT* | STY3100 | STM2962 | 1.385 | 8.70E-04 |
| *yfhF* | STM2541 | STM2541 | 1.385 | 4.51E-04 |
| *priB* | STY4748 | STM4392 | 1.384 | 6.39E-04 |
| *fliC* | STM1959 | STM1959 | 1.379 | 5.46E-04 |
| *feoB* | STY4290 | STM3506 | 1.379 | 3.08E-03 |
| *garK* | STM3247 | STM3247 | 1.373 | 2.71E-03 |
| *proQ* | STY1977 | STM1846 | 1.372 | 2.90E-03 |
| *yjcD* | STY4466 | STM4268 | 1.370 | 6.59E-05 |
| *guaB* | STM2511 | STM2511 | 1.368 | 1.04E-03 |
| *rpmC* | STY4366 | STM3432 | 1.363 | 3.40E-04 |
| *rimM* | STY2862 | STM2675 | 1.359 | 1.19E-03 |
| *rpsP* | STY2863 | STM2676 | 1.357 | 3.40E-03 |
| *dacA* | STY0688 | STM0637 | 1.354 | 3.86E-04 |
| *rplP* | STY4365 | STM3433 | 1.354 | 2.57E-04 |
|  | STM2754 | STM2754 | 1.352 | 1.12E-02 |
| *rplS* | STY2860 | STM2673 | 1.343 | 8.05E-04 |
| *rpsC* | STY4364 | STM3434 | 1.341 | 2.81E-03 |
| *iap* | STM2936 | STM2936 | 1.339 | 2.56E-03 |
| *pyrB* | STY4800 | STM4460 | 1.336 | 4.62E-04 |
|  | STM1585 | STM1585 | 1.335 | 6.40E-03 |
| *rplA* | STM4150 | STM4150 | 1.335 | 2.49E-04 |
| *purE* | STM0534 | STM0534 | 1.334 | 9.39E-06 |
| *secG* | STM3293 | STM3293 | 1.332 | 1.54E-03 |
|  | STY1982 | STM1851 | 1.330 | 2.36E-03 |
| *ygcY* | STY3099 | STM2961 | 1.330 | 7.11E-03 |
| *atpH* | STY3910 | STM3868 | 1.330 | 2.09E-03 |
| *rplR* | STM3424 | STM3424 | 1.329 | 1.42E-03 |
| *gcvP* | STM3053 | STM3053 | 1.329 | 1.94E-03 |
| *cyoA* | STY0485 | STM0443 | 1.328 | 1.39E-04 |
| *feoA* | STM3505 | STM3505 | 1.328 | 4.02E-04 |
| *fdx* | STY2784 | STM2538 | 1.327 | 1.58E-03 |
| *atpF* | STY3909 | STM3869 | 1.326 | 2.34E-03 |
| *pipA* | STY1115 | STM1087 | 1.325 | 1.71E-02 |
| *infC* | STM1334 | STM1334 | 1.324 | 3.19E-03 |
| *sdaB* | STM2971 | STM2971 | 1.322 | 3.15E-03 |
|  | STY2906 | STM2786 | 1.321 | 1.14E-02 |
| *tsf* | STM0217 | STM0217 | 1.321 | 3.88E-03 |
| *exbB* | STY3332 | STM3159 | 1.318 | 4.98E-03 |
| *gcvH* | STY3210 | STM3054 | 1.316 | 1.40E-02 |
| *sifB* | STM1602 | STM1602 | 1.310 | 9.16E-04 |
| *smpB* | STM2688 | STM2688 | 1.309 | 1.38E-02 |
| *accC* | STM3380 | STM3380 | 1.308 | 3.19E-04 |
| *rpsM* | STY4380 | STM3418 | 1.308 | 4.14E-03 |
| *yhdG* | STY3564 | STM3384 | 1.306 | 2.14E-04 |
| *trmD* | STY2861 | STM2674 | 1.303 | 1.31E-02 |
| *sbp* | STM4063 | STM4063 | 1.303 | 2.05E-03 |
| *rpmE* | STY3774 | STM4096 | 1.303 | 3.61E-03 |
| *atpB* | STY3907 | STM3871 | 1.301 | 1.96E-03 |
| *dsbA* | STY3883 | STM3997 | 1.301 | 2.93E-03 |
| *yciG* | STY1323 | STM1728 | -1.302 | 1.26E-03 |
| *sfbA* | STM0510 | STM0510 | -1.304 | 2.32E-03 |
|  | STM1635 | STM1635 | -1.304 | 4.85E-03 |
| *yohF* | STY2401 | STM2171 | -1.306 | 2.35E-03 |
| *yqjG* | STY3413 | STM3233 | -1.308 | 1.15E-02 |
| *prgI* | STY2994 | STM2873 | -1.310 | 2.15E-04 |
|  | STY0091 | STM0080 | -1.312 | 1.20E-03 |
| *narG* | STY1288 | STM1764 | -1.314 | 8.81E-03 |
| *phsC* | STM2063 | STM2063 | -1.314 | 5.52E-03 |
|  | STM2341 | STM2341 | -1.322 | 9.95E-03 |
| *aidB* | STM4377 | STM4377 | -1.325 | 8.78E-05 |
| *narU* | STM1576 | STM1576 | -1.326 | 1.59E-02 |
| *yqaE* | STY2916 | STM2796 | -1.326 | 1.73E-02 |
| *hisQ* | STM2353 | STM2353 | -1.327 | 8.67E-05 |
| *bolA* | STY0488 | STM0446 | -1.328 | 3.17E-03 |
| *yqjK* | STY3411 | STM3231 | -1.328 | 3.10E-03 |
| *yiaG* | STY4154 | STM3648 | -1.340 | 2.92E-03 |
| *blc* | STM4339 | STM4339 | -1.340 | 7.88E-03 |
| *csiE* | STY2800 | STM2553 | -1.342 | 5.71E-04 |
| *yajO* | STY0460 | STM0421 | -1.345 | 2.17E-03 |
|  | STY1787 | STM1324 | -1.345 | 1.33E-02 |
| *aceE* | STY0175 | STM0152 | -1.346 | 4.55E-03 |
| *osmE* | STM1311 | STM1311 | -1.347 | 2.23E-03 |
| *yhcO* | STY3544 | STM3363 | -1.352 | 3.78E-05 |
| *wraB* | STY1155 | STM1119 | -1.352 | 1.89E-03 |
| *ydeI* | STY1545 | STM1515 | -1.358 | 1.04E-03 |
| *fadL* | STY2623 | STM2391 | -1.359 | 1.17E-03 |
| *fic* | STM3470 | STM3470 | -1.362 | 9.63E-04 |
| *astA* | STM1304 | STM1304 | -1.363 | 3.23E-03 |
| *osmB* | STY1346 | STM1705 | -1.364 | 6.08E-04 |
| *yjiA* | STY4888 | STM4530 | -1.365 | 5.36E-03 |
| *ugpB* | STY4254 | STM3557 | -1.365 | 7.33E-04 |
| *ygaM* | STY2925 | STM2802 | -1.365 | 2.14E-05 |
| *yqhE* | STM3165 | STM3165 | -1.367 | 6.95E-03 |
|  | STM1078 | STM1078 | -1.368 | 1.72E-05 |
| *yghA* | STM3157 | STM3157 | -1.368 | 2.52E-03 |
|  | STY4912 | STM4562 | -1.368 | 2.69E-05 |
|  | STM0362 | STM0362 | -1.368 | 5.71E-03 |
| *yqjE* | STY3410 | STM3230 | -1.370 | 9.44E-04 |
| *sodC* | STM1440 | STM1440 | -1.380 | 7.70E-03 |
| *ybgS* | STM0759 | STM0759 | -1.382 | 2.21E-03 |
| *ybiH* | STM0819 | STM0819 | -1.382 | 1.30E-03 |
| *yhbO* | STY3452 | STM3269 | -1.383 | 2.68E-03 |
| *yhfG* | STM3471 | STM3471 | -1.384 | 4.84E-04 |
|  | STM2585 | STM2585 | -1.384 | 4.68E-03 |
| *clpA* | STM0945 | STM0945 | -1.386 | 4.97E-03 |
| *gltI* | STY0710 | STM0665 | -1.386 | 3.73E-03 |
| *prgJ* | STY2993 | STM2872 | -1.391 | 3.10E-04 |
|  | STY3293 | STM3124 | -1.392 | 6.09E-03 |
| *kdgT* | STY0183 | STM0161 | -1.396 | 7.99E-03 |
| *gcd* | STM0169 | STM0169 | -1.397 | 4.93E-03 |
|  | STM0948 | STM0948 | -1.397 | 3.79E-03 |
| *pfkB* | STM1326 | STM1326 | -1.401 | 2.08E-04 |
| *ompX* | STM0833 | STM0833 | -1.402 | 6.68E-03 |
| *yhaH* | STY3414 | STM3234 | -1.405 | 2.63E-03 |
| *yeeZ* | STY2279 | STM2070 | -1.407 | 4.63E-03 |
| *mscL* | STY4387 | STM3410 | -1.408 | 2.94E-03 |
| *narH* | STM1763 | STM1763 | -1.409 | 7.00E-03 |
| *narY* | STM1578 | STM1578 | -1.412 | 1.67E-03 |
|  | STM2698 | STM2698 | -1.416 | 8.48E-03 |
|  | STM1039 | STM1039 | -1.417 | 1.73E-02 |
| *yibF* | STY4112 | STM3684 | -1.419 | 2.79E-04 |
| *emrD* | STM3798 | STM3798 | -1.419 | 1.66E-03 |
| *yddX* | STY1496 | STM1564 | -1.424 | 4.65E-04 |
| *ycfS* | STM1215 | STM1215 | -1.426 | 3.91E-03 |
| *ydcW* | STM1597 | STM1597 | -1.430 | 5.50E-03 |
|  | STY4259 | STM3552 | -1.432 | 2.34E-03 |
|  | STM2591 | STM2591 | -1.432 | 6.37E-03 |
| *potF* | STM0877 | STM0877 | -1.434 | 1.11E-03 |
| *cycA* | STM4398 | STM4398 | -1.437 | 2.38E-03 |
| *tctD* | STM2785 | STM2785 | -1.442 | 8.92E-03 |
| *osmC* | STY1497 | STM1563 | -1.454 | 1.10E-03 |
| *psiF* | STM0384 | STM0384 | -1.457 | 1.40E-04 |
| *pagC* | STM1246 | STM1246 | -1.458 | 1.69E-03 |
| *ydiZ* | STM1325 | STM1325 | -1.458 | 2.25E-04 |
|  | STM0927 | STM0927 | -1.467 | 8.65E-03 |
| *yeaG* | STY1831 | STM1285 | -1.470 | 3.91E-04 |
| *poxB* | STM0935 | STM0935 | -1.477 | 3.28E-03 |
| *ybaY* | STY0509 | STM0465 | -1.482 | 8.12E-04 |
| *yceP* | STM1161 | STM1161 | -1.483 | 4.15E-03 |
| *galR* | STY3155 | STM3011 | -1.485 | 3.48E-03 |
| *tktB* | STY2711 | STM2474 | -1.486 | 1.66E-04 |
| *ybeF* | STY0684 | STM0634 | -1.487 | 3.05E-04 |
| *rsd* | STM4165 | STM4165 | -1.494 | 6.28E-04 |
|  | STY2647 | STM2406 | -1.500 | 2.57E-03 |
|  | STM0360 | STM0360 | -1.507 | 2.00E-03 |
| *talA* | STY2710 | STM2473 | -1.509 | 2.51E-04 |
| *narZ* | STM1577 | STM1577 | -1.512 | 7.95E-03 |
| *sipB* | STY3008 | STM2885 | -1.513 | 6.21E-03 |
|  | STM2405 | STM2405 | -1.517 | 2.94E-04 |
| *putA* | STY1159 | STM1124 | -1.523 | 7.09E-03 |
| *lldP* | STM3692 | STM3692 | -1.524 | 1.42E-03 |
| *hisJ* | STY2584 | STM2354 | -1.530 | 1.33E-03 |
| *ompC* | STY2493 | STM2267 | -1.534 | 8.04E-03 |
| *gabT* | STY2912 | STM2792 | -1.534 | 2.31E-03 |
| *ygdI* | STY3123 | STM2983 | -1.544 | 1.42E-03 |
| *yljA* | STM0944 | STM0944 | -1.546 | 4.21E-03 |
| *yeaH* | STM1284 | STM1284 | -1.553 | 1.00E-03 |
|  | STY2928 | STM2804 | -1.555 | 2.20E-04 |
|  | STY1971 | STM1841 | -1.561 | 5.39E-04 |
| *mglA* | STM2189 | STM2189 | -1.572 | 1.27E-03 |
| *sipC* | STM2884 | STM2884 | -1.586 | 1.61E-03 |
| *ygaU* | STM2795 | STM2795 | -1.594 | 1.54E-03 |
| *msrA* | STM4408 | STM4408 | -1.598 | 6.60E-05 |
| *ycgB* | STM1804 | STM1804 | -1.606 | 3.24E-03 |
| *acnA* | STM1712 | STM1712 | -1.610 | 1.16E-03 |
| *dppA* | STY4168 | STM3630 | -1.611 | 2.03E-04 |
|  | STY0853 | STM0818 | -1.615 | 2.71E-03 |
| *ompA* | STM1070 | STM1070 | -1.646 | 1.71E-03 |
|  | STM3012 | STM3012 | -1.656 | 4.31E-03 |
| *cstA* | STY0644 | STM0600 | -1.668 | 5.63E-05 |
| *yigI* | STY3603 | STM3956 | -1.676 | 1.05E-02 |
| *gabD* | STM2791 | STM2791 | -1.703 | 1.68E-04 |
| *fadD* | STY1948 | STM1818 | -1.704 | 9.99E-04 |
|  | STM1050 | STM1050 | -1.712 | 4.91E-04 |
| *spvA* | PSLT040 | PSLT040 | -1.740 | 8.57E-04 |
| *ygjU* | STY3405 | STM3225 | -1.765 | 9.54E-05 |
| *fimA* | STM0543 | STM0543 | -1.766 | 2.91E-04 |
| *argT* | STY2585 | STM2355 | -1.774 | 1.31E-06 |
|  | STM2609 | STM2609 | -1.781 | 2.22E-03 |
| *nmpC* | STM1572 | STM1572 | -1.791 | 1.92E-03 |
| *yjiJ* | STM4515 | STM4515 | -1.810 | 5.62E-05 |
| *acs* | STY4473 | STM4275 | -1.822 | 2.58E-04 |
| *yjcG* | STY4471 | STM4273 | -1.832 | 1.16E-04 |
| *oat* | STY3396 | STM3218 | -1.856 | 4.22E-05 |
| *yjcH* | STY4472 | STM4274 | -1.857 | 1.50E-04 |
|  | STM2590 | STM2590 | -1.894 | 9.02E-04 |
| *phoH* | STM1126 | STM1126 | -1.904 | 3.90E-04 |
| *gntT* | STM3512 | STM3512 | -1.921 | 1.27E-04 |
|  | STY0391 | STM0359 | -1.939 | 4.37E-05 |
| *mig-3* | STM1868 | STM1868 | -1.954 | 2.59E-03 |
| *gabP* | STY2913 | STM2793 | -2.018 | 1.55E-03 |
|  | STM1041 | STM1041 | -2.053 | 1.15E-05 |
| *galM* | STY0806 | STM0773 | -2.093 | 2.75E-04 |
| *ycaR* | STM0987 | STM0987 | -2.105 | 1.38E-03 |
| *ygaF* | STM2790 | STM2790 | -2.128 | 1.78E-03 |
| *rbsB* | STM3884 | STM3884 | -2.144 | 1.28E-04 |
|  | STM2592 | STM2592 | -2.344 | 3.02E-05 |
| *galS* | STM2191 | STM2191 | -2.458 | 3.03E-04 |
|  | STM2586 | STM2586 | -2.560 | 2.37E-04 |
|  | STM2789 | STM2789 | -2.603 | 4.86E-07 |
| *yjiX* | STY4889 | STM4531 | -2.665 | 1.93E-04 |
| *galP* | STY3244 | STM3091 | -2.732 | 5.60E-05 |
|  | STM2594 | STM2594 | -2.816 | 2.82E-05 |
| *galE* | STY0809 | STM0776 | -2.928 | 4.02E-04 |
|  | STM2595 | STM2595 | -2.982 | 1.91E-04 |
|  | STM2606 | STM2606 | -3.022 | 2.85E-04 |
| *galK* | STM0774 | STM0774 | -3.165 | 2.07E-06 |
|  | STM0926 | STM0926 | -3.248 | 1.74E-04 |
|  | STM2597 | STM2597 | -3.278 | 3.75E-03 |
| *galT* | STY0808 | STM0775 | -3.340 | 2.82E-05 |
|  | STM2593 | STM2593 | -3.411 | 3.96E-06 |
|  | STM1049 | STM1049 | -3.439 | 6.39E-05 |
|  | STM2589 | STM2589 | -3.671 | 2.42E-04 |
|  | STM2607 | STM2607 | -3.760 | 2.05E-05 |
| *ompF* | STY1002 | STM0999 | -3.761 | 1.94E-05 |
|  | STM2608 | STM2608 | -3.922 | 8.12E-04 |
|  | STM2587 | STM2587 | -4.059 | 1.07E-04 |
|  | STM2588 | STM2588 | -4.258 | 3.35E-05 |
|  | STM2596 | STM2596 | -4.375 | 1.11E-04 |
|  | STM2602 | STM2602 | -4.774 | 7.21E-04 |
|  | STM2598 | STM2598 | -5.043 | 4.31E-05 |
|  | STM2601 | STM2601 | -5.608 | 1.54E-04 |
|  | STM2611 | STM2611 | -5.636 | 3.73E-04 |
|  | STM2600 | STM2600 | -5.937 | 2.35E-04 |
|  | STM2603 | STM2603 | -6.482 | 4.36E-04 |
| *fljB* | STM2771 | STM2771 | -7.238 | 1.37E-06 |
|  | STM2604 | STM2604 | -10.615 | 2.29E-05 |
|  | STM2605 | STM2605 | -11.597 | 1.60E-05 |
| *yjiY* | STY4890 | STM4532 | -11.665 | 1.43E-03 |

^a^ STY prefix denotes the gene number on the *S*. Typhi CT18 genome;

^b^ STM prefix denotes the gene number on the *S*. Typhimurium LT2 genome.

**Table S2. Primers used in this study**

| **Primer** | **Sequence (from 5’ to 3’)** | **Purpose^a^** |
| --- | --- | --- |
| PRO254 | ATGAATTCCATATGAATATCCTCCTTAG | RV, M9 template |
| PRO336 | GCGGTGACCCGGGAGATCTGAATTC | FW, LM-PCR |
| PRO337 | GAATTCAGATC | RV, LM-PCR |
| PRO379 | ATACTAGTTAGTAAGTGTAGGCTGGAGCTGCTTC | FW, M9 template |
| PRO435 | TTATTCTCTGGTATAGTGCCGAATAAGGCATATGGAAGGATTCAGACATTGTGTAGGCTGGAGCTGCTTC | FW, D&W CMPG5624 |
| PRO436 | TTATTCGGAATGATGTGCTATTTTCTCTTGCTCACGGCGATACCAGTAATCATATGAATATCCTCCTTA | RV, D&W CMPG5624 |
| PRO473 | GGATCCGAATAAGGCATATGGAAGGATTC | FW, *fabR* amplification |
| PRO474 | GAATTCATTACTCGTCCTTCACGTTATTC | RV, *fabR* amplification |
| PRO494 | CGCCGTGAGCAAGAGAAAATAGCACATCATTCCGAAACCGAGCTCGGATCCACTAG | FW, CMPG5825 |
| PRO495 | TTTATCTTGACCTGACTGTTTCATTACTCGTCCTTCCATATGAATATCCTCCTTAG | RV, CMPG5825 |
| PRO0505 | CCGTTAGTGCCGCCCTTAG | FW, (ChIP-)q(RT-)PCR *dnaG* |
| PRO0506 | AGCCAAGTCCAGGCAGCTT | RV, (ChIP-)q(RT-) PCR *dnaG* |
| PRO0742 | TTCAGTCCCGTCAGTCAACATT | FW, ChIP-qPCR IR*lsrR-lsrA* |
| PRO0743 | ATGCAGTATTGTGACTGATTTGCA | RV, ChIP-qPCR IR*lsrR-lsrA* |
| PRO1150 | AAACGCGGGCAACTTCAG | FW, qRT-PCR *rfaH* |
| PRO1151 | GTCAGGCAACTTACCGCTTGT | RV, qRT-PCR *rfaH* |
| PRO1523 | TCGCGTTTATCTACCATGTTTTATG | FW, ChIP-qPCR IR*fabA*-*lonP* |
| PRO1524 | TGTTCAGCGTACACGTGTTAGCT | RV, ChIP-qPCR IR*fabA*-*lonP* |
| PRO1621 | CGCCCGCGATGACAA | FW, qRT-PCR *fabB* |
| PRO1622 | AAAAAGCGTCCCGTACCTATGA | RV, qRT-PCR *fabB* |
| PRO1623 | CGCTAAAGGGCCGCAACT | FW, qRT-PCR *fabA* |
| PRO1624 | ACGACGCGGTCCATCATC | RV, qRT-PCR *fabA* |
| PRO2497 | AAGCCCCAGCGGGATAAC | FW, qRT-PCR *lsrA* |
| PRO2498 | TGTTCGGCATTATTGAGTTTGATATT | RV, qRT-PCR *lsrA* |
| PRO2489 | GCTCTTCGGTGAGAAAGGTGTT | FW, qRT-PCR *pyrI* |
| PRO2490 | CAGCGAATCACTATCGGTCTGA | RV, qRT-PCR *pyrI* |
| PRO2526 | TCGTAGCCCATTTCAAAGCC | FW, qRT-PCR *ompA* |
| PRO2581 | CTTCATTCACAATGATGGCCC | RV, qRT-PCR *ompA* |
| PRO5185 | GCGAGCTCCATGGCATAGTTAC | FW, qRT-PCR *spvA* |
| PRO5186 | GCCGTGGTTCCTGAGCAA | RV, qRT-PCR *spvA* |
| PRO5203 | CGTGCGCAGGCAAGAGA | FW, qRT-PCR *hns* |
| PRO5204 | CAACTTCTAATTTTTCCAGCATTTCTTC | RV, qRT-PCR *hns* |
| PRO5205 | CATCGTTTGGGCCGGTACT | FW, ChIP-qPCR IR*ddg*-*yfdZ* |
| PRO5206 | ATGGCTCCTGTTTTTCTTTGCA | RV, ChIP-qPCR IR*ddg*-*yfdZ* |
| PRO5762 | CCTGAATAATCGTGTTTACCGATGT | FW, ChIP-qPCR IR*hpaR*-*hpaG* |
| PRO5763 | GCCCCTTGTTTCTGTTAAACTTTG | RV, ChIP-qPCR IR*hpaR*-*hpaG* |
| PRO5770 | GCCTCTAGCGTAACTGAGAATGG | FW, ChIP-qPCR IR*yqfA-yqfB* |
| PRO5771 | CGCGTGTTAGTTGAGTTTTTGTAATT | RV, ChIP-qPCR IR*yqfA-yqfB* |
| PRO5714 | CCTGGCAGCGGTGATCC | FW, qRT-PCR *ompF* |
| PRO5715 | AAATTTCTGCTGCGTTTGCG | RV, qRT-PCR *ompF* |
| PRO5813 | CGCGACCAGCGTTATTTTTC | FW, qRT-PCR *lsrF* |
| PRO5814 | TGCGCCCCCATTTCC | RV, qRT-PCR *lsrF* |
| PRO5815 | ACGCCGAAGACCGCTTTAA | FW, qRT-PCR *aceB* |
| PRO5816 | CAATCAACAGGGTCGCTTTGA | RV, qRT-PCR *aceB* |
| PRO5817 | TTGCGCACCGATTTAAAGTG | FW, qRT-PCR *yqfA* |
| PRO5818 | CGACAGCCAGCCCATTG | RV, qRT-PCR *yqfA* |
| PRO5821 | CGCTTCCGTTTCCGTCTATCT | FW, qRT-PCR *yjiY* |
| PRO5822 | CATGACCTTCTGGGCGATGT | RV, qRT-PCR *yjiY* |
| PRO5869 | CACTGCACGTTTCATTCAATACC | FW, ChIP-qPCR IR*fabB*-*mnmC* |
| PRO5870 | GTACGCTATTGTGCCAATCGAA | RV, ChIP-qPCR IR*fabB*-*mnmC* |
| PRO5944 | TTATGCCTGTCCCACATACAAATAGATGGCCAGGAAGTGGCACACGCTGCGTGTAGGCTGGAGCTGCTTC | FW, D&W 10324 |
| PRO5945 | ATGGTGCAAAAACCATTAATGACGCAGGGATATTCGCTGGCTGAGGAAATTCATATGAATATCCTCCTTA | RV, D&W 10324 |
| PRO5946 | CCGTCTAGACATCGTTATGAACGTTTCTCCGGAG | FW, *yqfA* amplification |
| PRO5947 | GCCGAGCTCCGCCGCCATCCGGCACGGGCATCCG | RV, *yqfA* amplification |
| PRO6530 | ATTCTAGACACGTGTTAGCTATCCTGCGTGCTT | FW, *fabA* amplification |
| PRO6531 | ATGAATTCTATGGGCTTTGCAAGCATGATACCG | RV, *fabA* amplification |
| PRO6532 | ATTCTAGAAATCGAAACTTAAAAATAGTGCGAA | FW, *fabB* amplification |
| PRO6533 | ATGAATTCTTAGCGACCTGTTTCATTGTTGATA | RV, *fabB* amplification |

^a^ FW: forward primer; RV: reverse primer; IR: intergenic region; LM-PCR: ligation-mediated PCR.
